# Predictive value of biochemical markers CRP, WBC, and total cholesterol for postoperative dry eye syndrome following phacoemulsification cataract surgery

**DOI:** 10.5937/jomb0-59665

**Published:** 2026-01-06

**Authors:** Yenan Wang, Xizhe Wang, Zhen Li, Huiqing Yang, Xuxiang Zhang

**Affiliations:** 1 Xuanwu Hospital, Capital Medical University, Department of ophthalmology, Beijing, China

**Keywords:** C-reactive protein, white blood cell count, total cholesterol, biochemical markers, cataract surgery, dry eye syndrome, predictive diagnostics, C-reaktivni protein, broj belih krvnih zrnaca, ukupni holesterol, biohemijski markeri, operacija katarakte, sindrom suvog oka, prediktivna dijagnostika

## Abstract

**Background:**

Dry eye syndrome is a common complication following phacoemulsification cataract surgery, potentially influenced by systemic biochemical factors. This study aimed to evaluate the predictive efficiency of three routinely measured biochemical markers - C-reactive protein (CRP), white blood cell count (WBC), and total cholesterol (TC) - in identifying patients at risk for postoperative dry eye syndrome.

**Methods:**

A total of 87 patients undergoing phacoemulsification between January 2024 and February 2025 were enrolled and categorized into dry eye (n=49) and non-dry eye (n=38) groups. Blood samples were collected preoperatively to assess CRP, WBC, and TC levels using standard laboratory protocols. Baseline characteristics were compared, and multivariate logistic regression was conducted to identify independent risk factors. Receiver operating characteristic (ROC) curves were generated to determine the predictive performance of each marker and their combination.

**Results:**

Patients in the dry eye group exhibited significantly elevated CRP, WBC, and TC levels compared to the non-dry eye group (P&lt;0.001 for all). Multivariate analysis identified CRP (O R = 12.679), WBC (O R = 3.216), and TC (OR= 1.258) as independent predictors. The area under the ROC curve (a Uc ) values for CRP WBC, and TC were 0.791, 0.770, and 0.757, respectively, while the combined model yielded an AUC of 0.936, indicating superior diagnostic performance (P&lt; 0.01).

**Conclusions:**

CRP, WBC, and TC levels are clinically accessible biochemical parameters that hold significant predictive value for dry eye syndrome following cataract surgery.Combined detection enhances prognostic accuracy andmay guide early intervention strategies to reduce postoperative complications.

## Abstract (sr)

**Uvod:**

Sindrom suvog oka je česta komplikacija nakon fakoemulzifikacione operacije katarakte, potencijalno pod uticajem sistemskih biohemijskih faktora. Cilj ove studije bio je da se proceni prediktivna efikasnost tri rutinski merena biohemijska markera - C-reaktivnog proteina (CRP), broja belih krvnih zrnaca (WBC) i ukupnog holesterola (TC) - u identifikaciji pacijenata sa rizikom od postoperativnog sindroma suvog oka.

**Metode:**

Ukupno 87 pacijenata koji su podvrgnuti fakoemulzifikaciji između januara 2024. i februara 2025. godine je uključeno u istraživanje i kategorisano u grupe sa suvim okom (n=49) i bez suvog oka (n=38). Uzorci krvi su prikupljeni pre operacije radi procene nivoa CRP WBC i TC korišćenjem standardnih laboratorijskih protokola. Osnovne karakteristike su upoređene i sprovedena je multivarijantna logistička regresija radi identifikacije nezavisnih faktora rizika. Generisane su ROC krive kako bi se odredila prediktivna efikasnost svakog markera i njihove kombinacije.

**Rezultati:**

Pacijenti u grupi sa suvim okom pokazali su značajno povišene nivoe CRP WBC i TC u poređenju sa grupom bez suvog oka (P&lt;0,001 za sve). Multivarijantna analiza je identifikovala CRP (O R = 12,679), WBC (O R= 3,216) i TC (O R = 1,258) kao nezavisne prediktore. Vrednosti površine ispod ROC krive (AUC) za CRP WBC i TC bile su 0,791, 0,770 i 0,757, respektivno, dok je kombinovani model dao AUC od 0,936, što ukazuje na superiorne dijagnostičke performanse (P&lt; 0,01).

**Zaključak:**

Nivoi CRP WBC i TC su klinički dostupni biohemijski parametri koji imaju značajnu prediktivnu vrednostza sindrom suvog oka nakon operacije katarakte. Kombinovana detekcija poboljšava prognostičku tačnost i možeusmeravati strategije rane intervencije za smanjenje postoperativnih komplikacija.

## Introduction

Cataract ranks among the leading causes of blindness worldwide. Clinically, phacoemulsification is the primary treatment modality, characterized by small incisions, rapid recovery, and high safety. However, postoperative complications remain a significant factor affecting patients' visual quality and overall quality of life. Among these complications, dry eye syndrome is particularly common, with a short-term incidence ranging from 30% to 60% and a long-term incidence still reaching 10% to 30% [1] [2]. Dry eye syndrome not only causes ocular surface discomfort and reduced visual quality but may also delay corneal healing, increase the risk of infection, and severely impact surgical outcomes [3].

C-reactive protein (CRP) is a sensitive marker of systemic inflammation and a common acute-phase reactant. White blood cell count (WBC) reflects immune defense and infection status, while total cholesterol (TC) is closely associated with lipid metabolism and vascular endothelial function [4] [5] [6]. Previous studies have shown that elevated levels of CRP and WBC are associated with chronic inflammation of the ocular surface, and abnormal TC levels may contribute to the development of dry eye syndrome by affecting meibomian gland lipid secretion [7]. However, the predictive value of combined CRP WBC, and TC levels for postoperative dry eye syndrome following phacoemulsification remains unclear. Based on the above, this study aims to explore the predictive efficacy of combined detection of CRP, WBC, and TC by observing their dynamic changes after surgery, providing a theoretical basis for early identification of high-risk patients and the development of individualized intervention strategies.

## Materials and methods

### General information

A total of 87 patients who underwent phacoemulsification at our hospital between January 2024 and February 2025 were included. Inclusion criteria: (1) age >18 years; (2) diagnosis of cataract according to the World Health Organization (WHO) criteria [1], confirmed by slit-lamp examination, fundus examination, and ocular ultrasound, with all patients having Grade III lens nuclear hardness; (3) eligibility for phacoemulsification surgery with no contraindications; (4) no prior diagnosis of dry eye syndrome; (5) no autoimmune diseases, and no medications affecting CRP WBC, or TC levels within the past three months. Exclusion criteria: (1) concurrent serious ocular diseases such as uveitis or glaucoma, or a history of previous ocular surgery; (2) use of anti-inflammatory or immunomodulatory drugs within the past three months; (3) pregnant or lactating women; (4) mental disorders.

Based on the development of dry eye syndrome postoperatively, patients were divided into a dry eye group (n = 49) and a non-dry eye group (n = 38). Written informed consent was obtained from all participants and their families before the study.

### Methods

Baseline data, including age, sex, and medical history, were obtained from the hospital's electronic medical records. Levels of CRP WBC, and TC were measured using standardized procedures. After an overnight fast and adequate rest, 5 mL of venous blood was collected from each patient. For CRP and WBC testing, EDTA anticoagulant tubes were used; for TC testing, plain serum tubes were used. CRP and TC were measured using Automatic Biochemical Analyzer with enzymatic colorimetric assay kits. WBC counts were analyzed using the impedance and flow cytometry technology. All samples were processed within 2 hours after collection. The laboratory participated in external quality assessment programs and implemented strict internal quality control using commercial control materials. Daily calibration and routine maintenance were conducted in accordance with the manufacturers' guidelines to ensure the precision and accuracy of all measurements.

### Observation indicators

(1) Comparison of baseline characteristics between the dry eye and non-dry eye groups.

(2) Comparison of CRP WBC, and TC levels between the two groups.

(3) Multivariate logistic regression analysis of factors influencing the development of dry eye syndrome post-phacoemulsification.

(4) Receiver operating characteristic (ROC) curves and area under the curve (AUC) calculations to evaluate the predictive value of CRP, WBC, and TC for postoperative dry eye syndrome.

### Statistical analysis

Data were analyzed using Statistic Package for Social Science (SPSS) 27.0 (IBM, Armonk, NY, USA). Measurement data were expressed as mean ± standard deviation (x̄ ± s), and independent samples t-tests were used for between-group comparisons. Categorical data were expressed as numbers and percentages (n (%)), and compared using the chi-square (χ^2^) test. ROC curves were plotted to assess the predictive value of CRP WBC, and TC. Multivariate logistic regression was conducted to identify risk factors for postoperative dry eye syndrome. A P-value < 0.05 was considered statistically significant.

## Results

### Comparison of baseline characteristics between the two groups

The dry eye group had significantly higher age, longer surgical duration, and a greater proportion of patients with a history of diabetes compared to the non-dry eye group (all P < 0.01). No statistically significant differences were observed in other baseline variables (P > 0.05). See Table 1.

**Table 1:** Comparison of baseline data between the two groups of patients (x̄±s), n (%).

Factor	Dry Eye Group (n = 49)	Non-Dry Eye Group (n = 38)	t (χ^2^) / P
Age (years, x̄±s)	62.06 ± 8.68	57.68 ± 8.65	12.178 / <0.001
Male (n, %)	34 (69.39%)	25 (65.79%)	0.127 / 0.722
BMI (kg/m^2^, x̄±s)	24.18 ± 2.46	24.29 ± 2.34	-0.204 / 0.839
Smoking history (n, %)	11 (22.45%)	10 (26.32%)	0.175 / 0.676
Alcohol consumption (n, %)	10 (20.41%)	9 (23.68%)	0.135 / 0.714
Hypertension history (n, %)	13 (26.53%)	12 (31.58%)	0.266 / 0.606
Diabetes history (n, %)	32 (65.31%)	10 (26.32%)	13.030 / <0.001
Duration of surgery (min, x̄±s)	11.94 ± 1.75	9.89 ± 1.35	5.954 / <0.001

### Comparison of CRP, WBC, and TC levels between the two groups

The levels of CRP WBC, and TC in the dry eye group were significantly higher than those in the non-dry eye group (all P < 0.001). See Table 2.

**Table 2:** Comparison of CRP WBC, and TC levels between the two groups (x̄±s).

Group	CRP (mg/L)	WBC (X10 /L )	TC (mmol/L)
Dry Eye Group (n = 49)	18.45 ± 1.11	6.90 ± 2.68	6.94 ± 2.38
Non-Dry Eye Group (n = 38)	10.50 ± 5.99	3.53 ± 1.50	4.40 ± 2.46
t/P	5.535 / <0.001	4.921 / <0.001	4.883 / <0.001

### Multivariate logistic regression analysis of risk factors for dry eye syndrome after phacoemulsification

Dry eye syndrome after phacoemulsification was set as the dependent variable, and variables with P < 0.05 in univariate analysis were included as independent variables. The assignment of variables is shown in Table 3.

**Table 3:** Variable assignment table.

Factor	Variable	Assignment
Age	X1	Entered as original value
History of diabetes	X2	Yes = 1, No = 0
Duration of surgery	X3	Entered as original value
CRP	X4	Entered as original value
WBC	X5	Entered as original value
TC	X6	Entered as original value
Postoperative dry eye syndrome<br>after phacoemulsification	Y	Yes = 1, No = 0

**Figure 1:**
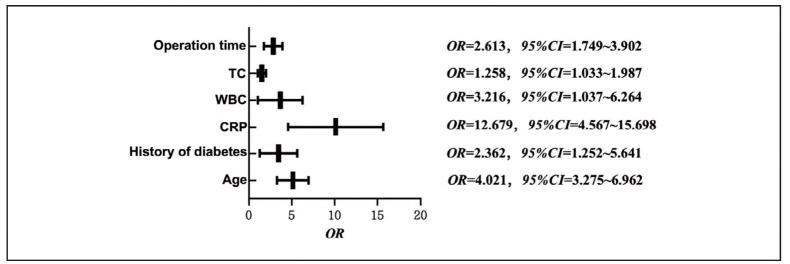
Multivariate logistic regression analysis of risk factors for dry eye syndrome after phacoemulsification.

Multivariate logistic regression analysis revealed that age, history of diabetes, duration of surgery, CRP, WBC, and TC were all risk factors for postoperative dry eye syndrome (OR = 4.021, 2.362, 2.613, 12.679, 3.216, 1.258; 95% CI = 1.033-15.698). See Table 4 and Figure 1.

**Table 4:** Multivariate logistic regression analysis of risk factors for dry eye syndrome after phacoemulsification.

Variable	β	S.E.	Wald	P	OR	95% CI
Age	1.649	0.457	3.608	<0.001	4.021	3.275-6.962
History of diabetes	2.109	0.506	4.168	0.006	2.362	1.252-5.641
Duration of surgery	0.960	0.205	22.011	<0.001	2.613	1.749-3.902
CRP	1.336	0.418	3.196	<0.001	12.679	4.567-15.698
WBC	1.862	0.463	4.022	<0.001	3.216	1.037-6.264
TC	2.175	0.527	4.127	<0.001	1.258	1.033-1.987

### Predictive Value of CRP, WBC, and TC levels for postoperative dry eye syndrome after phacoemulsification

**Figure 2:**
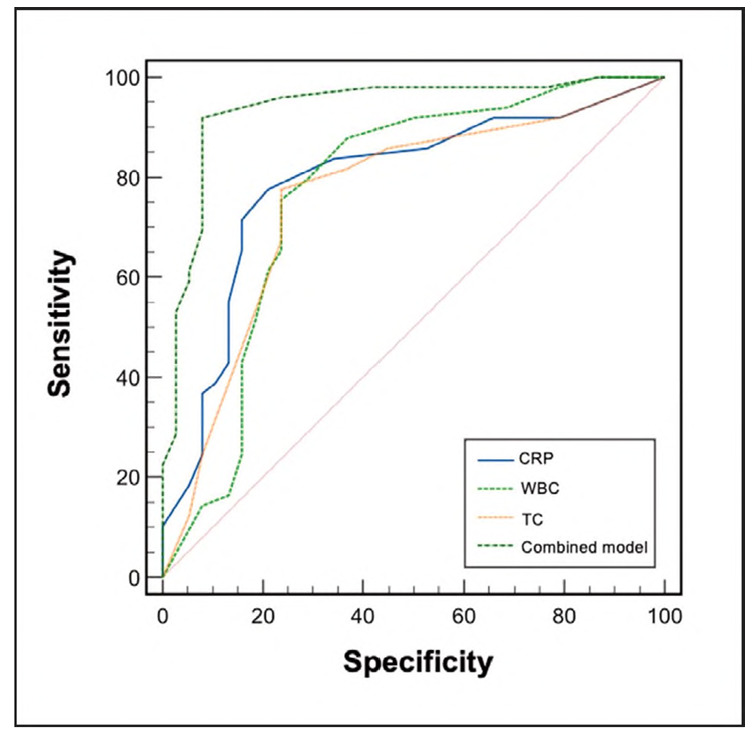
Predictive value of CRP WBC, and TC levels for postoperative dry eye syndrome after phacoemulsification.

ROC curve analysis showed that CRP, WBC, and TC levels had relatively high predictive value for dry eye syndrome after phacoemulsification (AUC: 0.791, 0.770, and 0.757, respectively). The combined detection model yielded a significantly higher AUC (0.936) than any single indicator (all P < 0.05). See Table 5 and Figure 2.

**Table 5:** Predictive value of CRP WBC, and TC levels for postoperative dry eye syndrome after phacoemulsification.

Indicator	AUC	S.E.	95%CI	Sensitivity	Specificity	Cut-off Value
CRP	0.791	0.051	0.690~0.870	77.55	78.95	>10
WBC	0.770	0.056	0.667~0.853	75.51	76.32	>9
TC	0.757	0.054	0.653~0.843	77.55	76.32	>5
Combined Model	0.936	0.029	0.863~0.977	91.84	92.11	-
Z/P_Combined Detection/CRP_	2.979/0.003	-	-	-	-	-
Z/P_Combined Detection/W BC_	2.761/0.006	-	-	-	-	-
Z/P_Combined DetectionAC_	2.746/0.006	-	-	-	-	-

## Discussion

The surgical incision in phacoemulsification can damage corneal nerves and reduce corneal sensitivity, thereby decreasing reflex tear secretion and blink frequency. A reduced blink rate not only impairs meibomian gland lipid secretion and accelerates tear evaporation, but also induces inflammatory responses and disrupts ocular surface structure. These multiple factors contribute to a vicious cycle of excessive tear evaporation, ultimately leading to dry eye syndrome [8] [9].

In this study, data showed that patients who developed dry eye syndrome postoperatively had significantly higher age, longer surgical duration, elevated CRP, WBC, and TC levels, as well as a higher proportion of diabetes history compared with those without dry eye. Multivariate logistic regression analysis confirmed these factors as independent risk factors for postoperative dry eye syndrome. Nur Faizah H et al. [10] reported a higher incidence of dry eye syndrome after phacoemulsification in cataract patients with diabetes. Bu Li et al. [11] suggested that elevated levels of inflammatory markers such as CRP in diabetic cataract patients may increase the risk of postoperative complications, including dry eye syndrome, supporting the rationale of the present study.

The underlying mechanisms may be as follows: With increasing age, lacrimal gland function declines, leading to decreased tear secretion and weakened repair capacity, thereby increasing dry eye risk [12]. Longer surgical duration prolongs ocular surface exposure, exacerbates corneal epithelial damage, and accumulates thermal injury due to phacoemulsification energy, compromising corneal nerves and tear film stability [13]. Chronic hyperglycemia induces corneal nerve degeneration and endothelial dysfunction, inhibits tear secretion, delays corneal epithelial healing, and further raises dry eye risk [14]. Elevated CRP and WBC levels indicate systemic inflammation, where inflammatory mediators can activate immune cells on the ocular surface, damaging corneal and conjunctival epithelial cells, impairing tear secretion, and aggravating tear film inflammation [15]. Hypercholesterolemia disrupts lipid metabolism and interferes with meibomian gland lipid secretion and composition. Abnormal lipid layers in the tear film accelerate tear evaporation and contribute to dry eye development [16].

Our results demonstrate that CRF, WBC, and TC each have a good predictive value for dry eye syndrome following phacoemulsification, with combined detection outperforming individual indicators. The likely reason is that CRP, as a sensitive marker of inflammation, reflects systemic inflammatory cascade activation and the upregulation of intercellular adhesion molecule-1 (ICAM-1), which promotes immune cell infiltration, damaging ocular surface tissues and disrupting tear film homeostasis. WBC count reflects immune defense status; increased neutrophils can release proteases and reactive oxygen species that harm corneal epithelial and goblet cells, affecting both tear secretion and tear film stability [17] [18]. Abnormal TC levels, linked to lipid metabolism disorders, may alter meibomian gland lipid composition, reducing tear film lipid layer fluidity and stability, thus accelerating tear evaporation [19].

The advantage of combined detection lies in the complementary nature of each biomarker: CRP identifies inflammatory activity, WBC quantifies immune response intensity, and TC reveals lipid metabolism abnormalities. This multi-parameter approach enables earlier detection of inflammation, assessment of immune-mediated tissue damage, and evaluation of tear film structure abnormalities, providing more precise identification of high-risk patients and a multidimensional basis for clinical early intervention.

This study is limited by its single-center design and relatively small sample size, which may affect the generalizability of the findings. Additionally, only short-term postoperative outcomes were evaluated, with no data on long-term follow-up. Future research should include larger, multicenter cohorts to enhance the robustness and applicability of the results. Extending the follow-up period will also provide a more comprehensive understanding of the temporal evolution and underlying mechanisms involved.

In conclusion, age, surgical duration, CRP, WBC, TC levels, and history of diabetes may all influence the development of postoperative dry eye syndrome after phacoemulsification. Among them, CRP, WBC, and TC demonstrate predictive value, with combined detection offering superior performance over single indicators. These factors may serve as a basis for early clinical prevention.

## Dodatak

### Conflict of interest statement

All the authors declare that they have no conflict of interest in this work.
